# Complete Genome Sequence of the Lumpy Skin Disease Virus Recovered from the First Outbreak in the Northern Caucasus Region of Russia in 2015

**DOI:** 10.1128/MRA.01733-18

**Published:** 2019-02-21

**Authors:** Alexander Sprygin, Yuriy Babin, Yana Pestova, Svetlana Kononova, Olga Byadovskaya, Aleksandr Kononov

**Affiliations:** a Federal Center for Animal Health, Vladimir, Russia; b Federal Budget Institution of Science, Central Research Institute of Epidemiology, Moscow, Russia; University of Arizona

## Abstract

We report here the complete genome sequence of a lumpy skin disease virus (LSDV) isolate obtained in the Northern Caucasus region of Russia in 2015. The LSDV/Russia/Dagestan/2015 genome sequence grouped with field LSDV isolates found in Serbia and Greece, suggesting the monophyletic origin of LSDV isolates that recently affected countries in the Northern Hemisphere.

## ANNOUNCEMENT

Lumpy skin disease (LSD) poses a tremendous threat to the international cattle industry, causing skin lesions, nasal and ocular discharge, fever, and a temporary drop in milk yield (1). The virus belongs to the Capripoxvirus genus of the *Poxviridae* family. Being endemic across wide areas of Africa in the past (1), LSD virus (LSDV) moved beyond its historic range and invaded Azerbaijan, the European Union, and Russia (2–4). Here, we report the complete genome sequence of an LSDV strain isolated from the first outbreak in the Dagestan Republic in 2015. The LSDV/Russia/Dagestan/2015 isolate, recovered from a skin lesion of an affected cow, was grown in ovine testis cells or goat gonad cells. DNA was purified from a 200 μl of a 10% resuspension of cell culture supernatant harvested when cytopathic effects were observed, using a QIAamp DNA minikit, according to the manufacturer’s recommendations (Qiagen, Germany) (5). Library preparations were performed using the Nextera XT DNA preparation kit (Illumina, USA) and sequenced on the MiSeq platform at the Federal Center for Animal Health (Vladimir, Russia).

For the genome assembly, reads were mapped to the reference genome (GenBank accession number AF409137) using Bowtie2 (6). The average read length was 60 bp. A total of 126,221 reads, making up 23.72% of the total reads, were mapped onto the reference sequence and used in the assembly. Paired-end reads were mapped onto the reference genome. Default Bowtie2 parameters were used to run the mapping. The consensus sequence was aligned with those of previously published LSDV genomes using BioEdit (7). For resolving discrepancies that were identified after manual inspection of the genome reference mapping, genome segments carrying mismatches were sequenced by Sanger sequencing. Open reading frames were predicted with the Genome Annotation Transfer Utility (GATU) (8) relative to the reference strain Neethling 2490. Discrepancies with previously published LSDV genomes were confirmed by Sanger sequencing. The quality of the raw data was assessed using FastQC with a Q score of >30. A total of 639,942 paired-end reads were mapped to the Neethling Warmbaths LW strain (AF409137), with an average coverage depth of 41.8× and an average map length of 250 nucleotides (nt). The Russia/Dagestan/2015 genome was 150,751 nt in length, with a genome organization identical to that of the reference strain Neethling Warmbaths LW (AF409137), containing 156 protein-coding genes, as predicted by the software.

Following genome assembly, the alignment with the complete LSDV genomes available in GenBank revealed that at the nucleotide level, the strain shares 99.8% nucleotide identity with the Neethling Warmbaths LW strain (GenBank accession number AF409137) and 99.9% nucleotide identity with the recently sequenced LSDV strains SERBIA/Bujanovac/2016 (9) and Evros/GR/15 strain (GenBank accession number KY829023) (10). Compared to the Neethling Warmbaths LW strain (GenBank accession number AF409137), four single nucleotide polymorphisms (SNPs) and two indels were identified and confirmed using the primers in Table 1.

**TABLE 1:** Primers used for confirmatory sequencing

Gene or region	Mutation	Primer	
		Forward	Reverse
LD076	C→T	GTTTGATCGTCAACTTCTAAATTTTG	CAAATTTATAGACCACGACATTTTT
LD128	G→A	AACACCCTATAAGGGACATGGT	GGACAATACATGATAGGATTAAACACA
LD128	T→A	TCCATTTGATTTTATTTCACCACA	TTTACCATGTCCCTTATAGGGTGT
Intergenic region between LD145 and LD146 genes	T→A	AACTCATAACTAAGAGGCGATATAAA	CATTAAAACATATTGATACATCGCAGT
LD022	ACTATT insertion	GTAATACATTACTTTATGAGGTGTCTTTC	CTGATAGTAAGTGAGGAAAAATGGA
LD141	3-bp deletion	CCTAAAGTACCCATTTGTTCAAGAGAC	TTCACCGTTTCCTCTCCATC

SNPs were found in the intergenic region between the LD145 and LD146 genes; two SNPs were found in the gene LD128, whereas the other one was detected in the gene LD076. Both changes in the LD128 gene were nonsynonymous, which led to amino acid substitutions in the corresponding proteins. The two indels, typical of the LSDV strains from Serbia and Greece (10), were coding genes and caused either the elimination of a single amino acid (LD141) or shifts in the reading frame of the encoded protein (LD022).

### Data availability.

The complete genome sequence of the LSDV isolate LSDV/Russia/Dagestan/2015 has been submitted to GenBank under accession number MH893760. Raw sequence data have been submitted to the SRA under BioProject number PRJNA515191.
